# HEALTH AND SOCIAL CARE TRAINEES’ ATTITUDES ABOUT AGING AND OLDER ADULTS

**DOI:** 10.1093/geroni/igad104.3379

**Published:** 2023-12-21

**Authors:** Min-Kyoung Park, Diane Martin

**Affiliations:** University of Maryland Baltimore and Baltimore County, Baltimore, Maryland, United States; University of Maryland, Baltimore, Baltimore, Maryland, United States

## Abstract

Ageism is pervasive in healthcare. It costs billions of healthcare dollars and results in earlier patient mortality and decreased quality of life. Existing research highlights the prevalence of ageist attitudes among health and social care practitioners, but there is a dearth of research regarding attitudes of trainees toward older adults. The present study utilized the Fraboni Scale of Ageism to examine attitudes of health and social care trainees participating in geriatric interprofessional training programs. Participants completed an electronic survey prior to the didactic portion of the program. Seventy-seven surveys were completed between November 2022 and June 2023. Results revealed a nuanced understanding of older adults’ contributions, particularly regarding creativity, social and political engagement, and the richness of their accumulated life experiences. Positive sentiments were evident regarding interactions and participants acknowledged the self-management capacities of older adults, encompassing personal hygiene and other aspects of independent living. Notably, findings underscore substantial support for creation of inclusive spaces. Overall, a person-centric understanding of older adulthood was evident in the results; less than 20% of trainees expressed a negative perception of older adults/later life to any given question. Recognizing the significance of bridging intergenerational perception disparities to counter ageism, this research contributes to a foundational understanding of younger generations’ awareness of aging. Given the contrast to existing research regarding attitudes of practitioners toward older adults, these results emphasize the importance of fostering opportunities for intergenerational dialogue and activities to maintain an attitude of inclusivity and empathy that underscores a person-centered approach to healthcare.

